# Cardiac MR for ventricular scar evaluation in patients with implanted defibrillators: technical and safety issues, and initial results

**DOI:** 10.1186/1532-429X-11-S1-P70

**Published:** 2009-01-28

**Authors:** Harold Litt, Francis Marchlinski

**Affiliations:** University of Pennsylvania School of Medicine, Philadelphia, PA USA

**Keywords:** Late Enhancement, Catheter Ablation Procedure, Late Enhancement Image, Refractory Ventricular Arrhythmia, Scar Mapping

## Introduction

Catheter based RF ablation is becoming an increasingly accepted treatment for refractory ventricular arrhythmias and late enhancement MRI can assist by providing anatomic maps of regions of scar which may form the basis for the arrhythmia. Unfortunately, many patients with ventricular arrhythmias have defibrillators (ICDs) implanted early in the course of their disease, prior to MR imaging assessment of cardiac structure, function, and scar burden. As ICDs are generally considered a contraindication to MRI, and the presence of a defibrillator may cause significant artifacts in the chest even if a study is performed, the use and utility of cardiac MR has not been studied in these patients.

## Purpose

To evaluate the feasibility and safety of performing cardiac MR in patients with ICDs and refractory ventricular arrhythmias and provide an initial assessment of its utility for scar mapping related to RF ablation procedures.

## Methods

Six patients with ICDs suffering from refractory ventricular tachyarrhythmias were imaged within several days of catheter ablation procedures. All patients gave informed consent for performing MRI in the presence of an ICD. The devices were interrogated prior to and after the study; tachycardia detection and therapies were turned off, and the pacemakers were set to VVI mode at a rate of 40 bpm for the duration of the MRI.

All studies were performed at 1.5 T. When possible, low SAR non-balanced gradient-echo cine and IR-prepped late-enhancement sequences were used, however real-time SSFP sequences were used in the presence of frequent ventricular ectopic beats. Imaging parameters were adjusted to minimize artifacts from the device.

## Results

All studies were completed safely, no patients reported any discomfort, and there were no episodes of tachycardia requiring treatment. Post-procedure device parameters, including thresholds, lead impedances, and battery voltage were unchanged in all patients, and all devices could be reprogrammed to previous settings. Real time SSFP cine sequences were needed in two patients with frequent ventricular ectopic beats. As expected, spin echo sequences were less affected by artifact from the ICD generator than gradient echo sequences, with SSFP cine sequences showing greater artifact than non-balanced gradient echo cine sequences. Late enhancement images were particularly affected, with significant difficulties encountered in nulling the myocardium in regions affected by artifact. Increasing bandwidth, with resultant decrease in TE, decreased the size of the device related artifact but also impacted SNR. The degree to which cardiac evaluation was limited by artifact was most influenced by the size of the patient and distance from the generator to the left ventricle.

**Figure 1:**
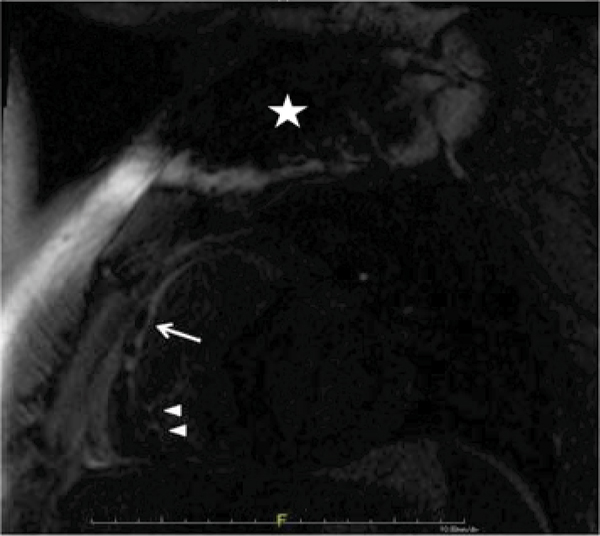
Figure 1

Two patients were diagnosed with right ventricular cardiomyopathy with good visualization of RV structure, function, and scar. One patient had hypertrophic cardiomyopathy, and three non-specific LV cardiomyopathies, with variable limitations in visualization of the LV antero-lateral wall and apex. All patients did have evidence of focal myocardial scar in regions not affected by artifact; imaging information was consistent with electrophysiologic observations and was considered clinically valuable in all cases. Figure 1 shows a large region of scar in the RV free wall and RVOT (arrow) in a patient with RV cardiomyopathy; note pacemaker/ICD leads (arrowheads) and artifact from generator (star).

## Conclusion

The results of this initial study suggest that cardiac MR for scar mapping may be performed safely in patients with ICDs under certain circumstances, giving clinically relevant results in those with refractory ventricular arrhythmias having RF ablation procedures.

